# Motor Function Relating to the Accuracy of Self-Overestimation Error in Community-Dwelling Older Adults

**DOI:** 10.3389/fneur.2020.599787

**Published:** 2020-11-30

**Authors:** Tsubasa Kawasaki, Ryosuke Tozawa

**Affiliations:** ^1^Institute of Sports Medicine and Science, Tokyo International University, Kawagoe, Japan; ^2^Department of Physical Therapy, Faculty of Health Science, Ryotokuji University, Urayasu, Japan

**Keywords:** community-dwelling elderly, self-estimation error, motor function, walking ability, mobility

## Abstract

**Background:** Older adults often overestimate their motor performance, which represents a serious safety hazard. The cause of this self-overestimation is to date, not yet fully established. Thus, the present study aimed to reveal the factors associated with self-overestimation by focusing on motor function.

**Methods:** This study included 105 community-dwelling older adults [20 males, median (25, 75 percentile) age: 73.00 (69.50, 77.50)]. Participants were assessed for errors in their self-estimation using a two-step test. They estimated the two-step distance that could be reached with maximum effort. Thereafter, they performed the actual two-step action. Participants were comprehensively assessed for motor function by various tests (i.e., 10-meter Walking Test, Timed Up and Go Test, postural stability, and muscle strength). They were then divided into two groups (the self-underestimation or self-overestimation group) and their motor performances were compared. Multiple linear regression analysis was then utilized to investigate the relationship between self-estimation error and motor function.

**Results:** Significant differences were found between the two groups regarding age, weight, actual two-step distance, and the time required for the Timed Up and Go Test and 10-meter Walking Test (*p* < 0.05). The regression analysis showed that self-estimation error was significantly related to the result of the 10-meter Walking Test (beta = 0.24, *p* = 0.011).

**Conclusions:** The self-overestimation of motor performance, which is likely to lead to several dangers (i.e., falling or obstacle collision), was related to walking ability. Consequently, the results showed that the 10-meter Walking Test would assist in detecting the self-overestimation of motor performance.

## Introduction

Executive function is one of the frontal brain functions, which consists of motor prediction, working memory, dividing attention, and shifting mental sets, among others (1), thereby optimizing how goal formulation, action planning, goal-directed plans, and monitoring are carried out (2). These brain functions enable efficient action to be performed without creating accidents such as falling or colliding with obstacles.

Action planning is directly associated with falling risk or a fear of falling. A previous study showed that incongruence between action planning (the self-estimation of one's motor performance) and actual motor performance is an important cognitive-related factor (3) in the risk of falling. In particular, self-overestimation of one's performance (where estimated own performance > actual performance) is more problematic than self-underestimation. Individuals who consider that their performance is higher than it really, tend to take on impossible tasks. Sakurai et al. have reported that healthy young adults and non-fall-experienced older adults showed self-underestimation in stepping over an obstacle and that even older adults who had experienced falls showed a self-overestimation in the task. This suggests that the self-overestimation of one's motor performance was related to falling risk (4). Similar findings have also been shown by the reach forward test in patients with Parkinson's disease (PD) (5). Based on these previous reports, self-overestimation is regarded as one of the main causes of falling because self-estimation error disrupts an individual from taking appropriate action (6).

To date, the most reasonable and likely cause of self-overestimation is being unaware and unable to recognize the decline in one's motor performance due to aging and disease. In previous studies, self-overestimation in the reaching forward task showed low performance in terms of the reach distance in older adults (3, 7) and PD patients (5). This finding was also observed in the step-over test (4). In addition, Kawasaki et al. have reported that self-estimation error using the two-step test (TST) was correlated with actual performance in PD patients. Considering these studies, there is growing evidence that people with self-overestimation show actual motor performance, and it can be assumed that self-overestimation occurred because of a lack of self-recognition of one's own declining motor performance.

Previous studies have not assessed alternative motor tasks pother than the motor tasks (reach forward or step over) that were investigated in the measurement of self-estimation error [the only report is that self-overestimation is related to general motor performance in PD patients (8)]. The relationship between self-overestimation and decreased actual motor performance can be obtained by motor task specificity has not yet been fully investigated. It has not yet been established which motor function is associated with self-overestimation. Thus, it is necessary to investigate whether various motor functions are related to self-overestimation and the influence of motor task specificity.

The purpose of the present study is to reveal the relationship between self-overestimation and decreased actual motor performance by measuring performance for several motor tasks in older adults. The data from the investigation in older adults is useful for patients with diseases related to aging. To assess motor performance, representative tools that directly reflect moving ability (walking speed, postural stability, and muscle strength of lower extremities) were used. Additionally, the TST, which we have used in previous research (8), was used for assessment of the estimation error. This study hypothesizes that self-overestimation is associated with multiple motor performance functions and that there is a possibility that a task-specific relationship exists between self-overestimation and actual motor function.

## Methods

### Participants

One hundred and five healthy community-dwelling older adults (twenty males and eighty-five females) participated in this study. Of the 105 participants, 69 were categorized as young-older, and 36 were old-older. The median (25, 75 percentile) age of the participants was 73.0 (69.5, 77.5) (early older: 70.0 (67.0, 72.0), old older: 79.0 (76.0, 81.0). There were no participants with cognitive impairments which was measured by a six-item dementia screening tool (9). Exclusion criteria were: (a) having back or leg pain or a limited range of motion that could influence walking ability or thei ability to complete the TST (a-TST); and (b) that visual impairment could influence walking ability in the a-TST (the details of the a-TST is stated below). The tenets of the Declaration of Helsinki were followed, and participants provided informed consent before participating in the study. This study was approved by the Ryotokuji University Scientific Ethics Committee (approval, 2829).

### Measurement of Self-Estimation Error

**Figure 1:**
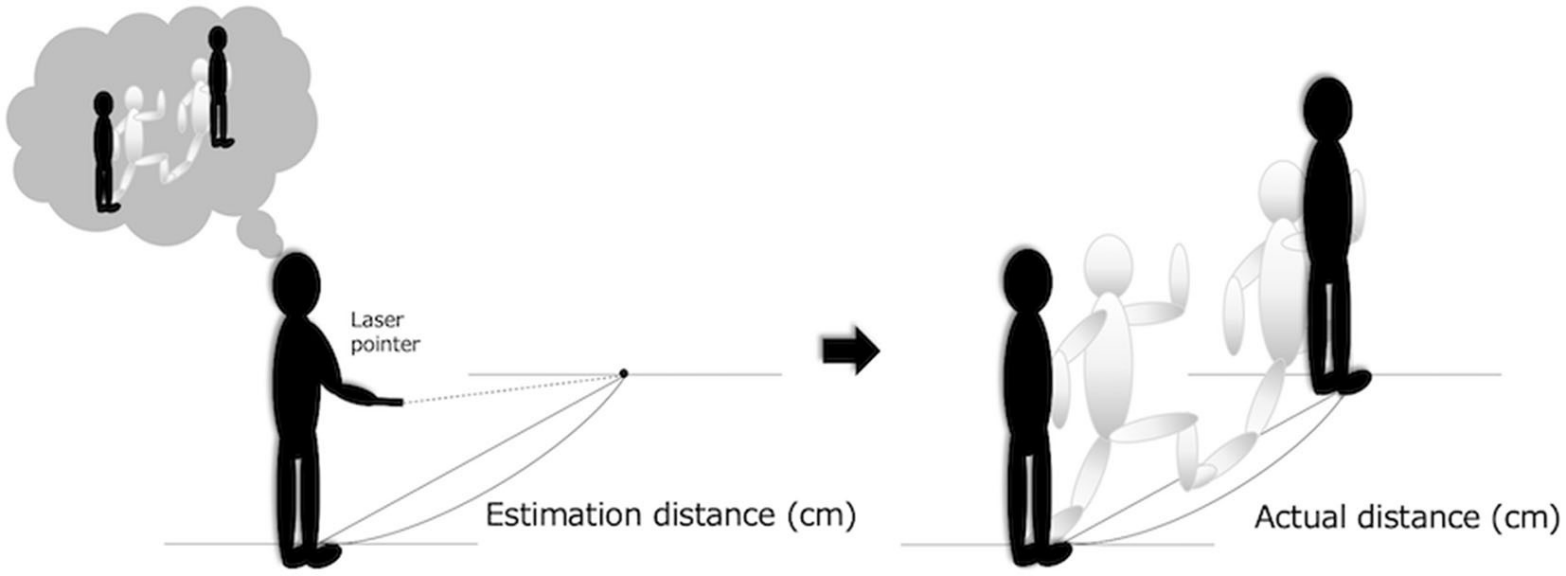
The estimation distance is the distance from toes to the predicted point. The actual distance is the distance from the starting point to the point reached in two steps. The difference between the two distances was used as an estimation error distance.

The measurement of self-estimation error was conducted following the method by Kawasaki et al. (8) (Figure 1). First, participants used a laser pointer to estimate the location of where two steps would lie had they employed their maximum effort to take these two steps (e-TST). The distance between participants' toes and the pointed location was defined as the self-estimation distance. Then, the actual distance of where two steps forward would lie was measured. The measured distance of the actual two steps was defined as the actual distance. In the a-TST, participants were asked that they (a) step toward their preferred side (right or left) in the initial step, (b) refrain from jumping, (c) stand still with both feet together before and after taking the two steps, and (d) do not look at the estimated point. Following the previous study (8), since the experience of taking two steps may affect the estimation of distance, a practice session was not conducted, and the estimation and actual trials were only carried out once. The self-estimated distance and actual distance were multiplied by individual height to cancel the influence of height. The self-estimation error was calculated by using the formula: value of e-TST minus a-TST.

### Measurements of Motor Function

Motor function was measured using tests to assess multiple motor abilities, i.e., walking ability, postural stability, and the muscle strength of the lower extremity. Walking ability was assessed by using the Timed Up and Go test (TUGT) (10) and 10-meter Walk Test (10MWT) (11). Postural stability was measured by the One-leg Standing test (OLST) (12) and Functional Reach Test (FRT) (13). Muscle strength was measured using the Stand-up test (SUT) (14). All assessments were conducted three times. The first assessment was performed to familiarize the participants with the test. The better of the two values from the last two assessments were used as the dependent measure.

Of these motor function assessments, in clinical and experimental settings, 10MWT and FRT have been widely regarded as routine assessments for individuals' walking and postural stability, respectively. 10MWT was executed by measuring the time required to walk 10 meters starting at the rater's cue. Considering a previous report, which showed that systematic error was not found in the maximum speed condition but not comfortable speed condition (15), participants walked at maximum speed in a 10 meter straight line, allowing 2 m acceleration and 2 m deceleration. The FRT was also executed by measuring the maximum distance of the reach of both arms when held forward while the participant stood in a fixed position. When the participants stepped forward during the trial, the assessments were performed again.

TUGT and OLST were used to diagnose musculoskeletal ambulation disability symptom complex that is a type of musculoskeletal disease disability (e.g., hip or knee osteoarthritis, osteoporosis, leg amputation) (16). TUGT was conducted by measuring the time taken to go from a sitting to standing position from a 40-cm height chair, walk 3 meters, turn around, walk 3 meters back, and sit on the chair. The participants performed the task as fast as possible, starting at the rater's cue. The reason for use of a fast speed was to exclude systematic errors based on a previous study, which showed that systematic TUGT errors were not found only in the fast-speed conditions (15). OLST was done by measuring how long the participants could stand upright beginning at the rater's starting cue. The participants crossed their arms in front of their chest, and the standing side (right or left) was optional. The maximum performance was set at 60 s, and when the participants reached 60 s, they were instructed to quit standing on one leg.

Additionally, the SUT and a-TST were also used in the diagnosis of the locomotive syndrome that is transferred because of musculoskeletal accidents (e.g., hip or knee stiffness, pain, limitation of range of motion) (17). The SUT was performed in the following manner: participants were instructed to stand up from four seats of different heights (40, 30, 20, and 10 cm) and to maintain the standing posture for at least 3 s using one or both legs once. The judgment of the SUT score was 0 to 8 and was allocated based on the height of the seat in a successful trial, depending on the trial difficulty, as reported by Ogata et al. (14) (Table 1).

**Table 1:** Scoring schema of the Stand-up Test.

	**Seat height (cm)**	**Rating score**
Both-leg	40	1^†^
	30	2
	20	3
	10	4
One-leg	40	5
	30	6
	20	7
	10	8

† *In case which failed 40 cm with both legs was rated score 0*.

### Statistical Analyses

To investigate the difference between participant characteristics and motor performance between both groups (self-overestimation and self-underestimation), the Pearson chi-square test (for sex), the Mann-Whitney U test (for age, height, and values in OLST, SUT), and unpaired *t*-test (for weight, and values in TUGT, 10MWT, FRT, a-TST) were performed depending on the scale type and distribution of the data. Then, to reveal the relationship between estimation error and motor function, a stepwise multiple linear regression analysis was conducted on the estimation error distance as the dependent variable. Dependent measures that showed group differences in comparisons (unpaired Student *t*-test or Mann–Whitney *U*-test) were used as independent variables. All statistical analyses were performed using SPSS for Windows (version 25.0; SPSS Inc., Chicago. IL, USA) and the statistical significance was set at *p* < 0.05.

## Results

The results showed the mean values of e-TST and estimation error as 1.21 ± 0.22 and −0.14 ± 0.19, respectively. Table 2 shows the results of the participants' characteristics and motor functions. There were significant differences between the groups (self-overestimation vs. self-underestimation) in age, weight, and three test values (TUGT, 10MWT, and a-TST). To illustrate this, the self-overestimation group was significantly slower in the time required for the TUGT and 10MWT and the value of a-TST was significantly shorter when compared to the self-underestimation group. Based on the results of the between-subject comparisons, five dependent variables (age, weight, 10MWT, TUGT, and a-TST) were inserted in the stepwise multiple linear regression analysis (note that the age and weight were regarded as confounders). These results showed that only the 10MWT was significantly related to self-estimation error (the standardized partial regression coefficient (*b*): 0.244, *p* = 0.013). The coefficient of determination for the generated model (adjusted *R*^2^) was 0.05 (*p* = 0.013) and multicollinearity was not shown (all variance inflation factor < 1.00).

**Table 2:** Characteristics and motor functions of the participants (overall) and in the two groups (self-overestimation and self-underestimation).

	**Overall (*N* = 105)**	**Underestimation (*N* = 85)**	**Overestimation (*N* = 20)**	***p*-value (two-tail)**
Gender (male/female)	21/84	16/69	5/15	0.542 ^*^
Age (years)	73.00 (69.50, 77.50)	72.00 (69.00, 76.00)	78.00 (71.50, 80.00)	0.006 ^†^
Height (cm)	155.0 (150.65, 159.00)	155.00 (151.00, 159.00)	155.25 (150.75, 159.75)	0.756 ^†^
Weight (kg)	54.00 ± 8.79	54.83 ± 8.41	50.50 ± 9.70	0.047 ^‡^
TUGT (sec)	6.89 ± 1.40	6.76 ± 1.35	7.45 ± 1.51	0.047 ^‡^
10MWT (sec)	5.71 ± 1.04	5.61 ± 1.04	6.16 ± 0.98	0.034 ^‡^
OLST (sec)	30.00 (15.18, 60.00)	30.42 (16.35, 60.00)	21.32 (8.37, 50.43)	0.144 ^‡^
FRT (cm)	29.84 ± 5.82	30.21 ± 5.95	28.32 ± 5.12	0.194 ^‡^
SUT	4.00 (3.00, 5.00)	4.00 (3.50, 5.00)	4.00 (3.00, 4.00)	0.065 ^†^
a-TST	1.35 ± 0.17	1.36 ± 0.16	1.27 ± 0.18	0.038 ^‡^

Data are shown as means ± standard deviation, or median (25, 75 percentile).

Two group comparisons by

* Chi-square test;

† Mann–Whitney U-test;

‡ Unpaired Student t-test.

*TUGT, Timed Up and Go Test; 10MWT, 10-meter Walk Test; OLST, One-leg Standing Test; FRT, Functional Reach Test; SUT, Stand-up Test; a-TST, Actual Two-step Test*.

## Discussion

The present study was undertaken to determine (a) whether self-overestimation is truly attributed to declining motor function, and (b) if so, which motor functions are related to self-overestimation. To investigate this, we conducted several motor function related assessments. The results of these tests showed that, in comparison to participants with self-underestimation, participants with self-overestimation showed lower motor performance in the a-TST, TUG, and 10MWT, but no difference in the FRT, OLST, and SUT. Thus, participants with self-overestimation showed low mobility in the a-TST, TUG, and 10MWT. This further suggests that self-overestimation of one's motor performance is associated with declining mobility rather than balance ability, as reflected in the FRT and OLST, or muscle strength as reflected in the SUT.

In particular, the self-estimation error was only related to the score obtained in the 10MWT as an explanation variable, as identified by the regression analysis. This result supported previous findings that showed a relationship between self-overestimation and walking ability, as mentioned above. Furthermore, this relationship can easily be understood by considering the cognitive functions common to “motor estimation” and “walking ability.” As a feature of self-estimation of one's motor performance, during the motor performance self-estimation, participants were involved in higher cognitive processes such as goal setting and motor planning (18). In previous studies, changes in cognitive ability were likely to lead to decreased walking ability. This relationship has been reported in a systematic review and meta-analysis of previously conducted cross-sectional studies (19). Additionally, a systematic review and meta-analysis in a longitudinal study (20) found that declining walking ability was a predictor that individuals may suffer from cognitive impairments, such as Alzheimer's disease (21), in the future. In particular, when investigating changes in motor function and cognitive ability in older adults, walking speed was correlated with a composite cognition, including executive functioning compared with muscle strength, lower-extremity functioning, or postural stability (20). These previous studies suggest that walking ability and cognitive function are correlated and could potentially suggest that a causal relationship exists. Furthermore, since cognitive processes are used during motor estimation, the relationship between estimation error and walking performance were reasonable findings.

Alternatively, differences in the a-TST value between participants with self-overestimation and self-underestimation may be explained by the decrease in their multiple motor functions. For instance, the a-TST asked participants to take two steps (which were as long as possible), which required several motor functions such as a wide range of motion at the hip joint, high levels of muscle strength, and high levels of postural stability. A-TST performance takes into account various motor functions, which is why it is used as one of the tools for detecting mobility disorder due to musculoskeletal accidents (i.e., locomotive syndrome), mainly in Japan (17). Our previous study investigating the relationship between estimation error and PD-related symptoms reported that the unified PD rating scale part III (cf. this shows general motor performance in PD patients) was correlated with estimation error (8). Since the a-TST placed a demand on general motor function, the present results showed that the difference in the a-TST performance between the two groups were similar to the findings in the PD data. However, because the a-TST is used to measure self-estimation error, the results were likely to include the impact of task specificity. This means that there were concerns regarding the robustness of the results; therefore, further studies are needed to verify this finding by including a general assessment of motor performance, e.g., Short Physical Performance Battery (22) or Motor Fitness Scale (23).

These findings on the relationships between overestimation and walking ability or general motor performance help to advance understanding of falling risk in older adults, building on the results of previous studies (4, 5, 7). Essentially, an increase in falling risk due to overestimation may mediate such motor performance.

There were several limitations to this study. Firstly, the cross-sectional design of the study inhibited its ability to distinguish causal relationships between self-overestimation and walking ability. Thus, future research should implement a longitudinal study to investigate whether there is a causal relationship. Secondly, this study lacked several assessments: sensory (superficial and deep sensory) or psychological functions [e.g., self-efficacy (24), apathy (25), and vitality (26)]. This could affect the low coefficient of determination in the regression analysis. Furthermore, since the self-estimation error may also be associated with alternative factors besides motor function, it is necessary to further investigate these factors. Lastly, since the 10MWT is a useful clinical assessment, the 10MWT may have availability for marker estimation error detection; however, further studies are required to further validate this point.

In conclusion, our findings, which add to existing literature on self-overestimation in older adults, indicate that the self-overestimation of one's motor function is not task-dependent. Furthermore, it is associated with a declining walking ability such as gait speed. These suggest that (i) self-overestimation could be subject to early detection by using the 10MWT, which is already a routine assessment in various settings, and (ii) that some interventions for self-efficacy (27) and social engagement (28) improve mobility and physical activity, and as a result, it may be clinically feasible to prevent overestimation.

## Data Availability Statement

The datasets generated for this study are available on request to the corresponding author.

## Ethics Statement

The studies involving human participants were reviewed and approved by the Ethics Committee of Ryotokuji University (approval, 2829). The patients/participants provided their written informed consent to participate in this study.

## Author Contributions

TK contributed with study concept and design of the survey, acquisition of data, analysis and interpretation of data, and drafting and revising the manuscript. RT contributed with study concept and design of the survey, acquisition of data, interpretation of data, and revising the manuscript. Both authors reviewed and approved the final manuscript.

## Conflict of Interest

The authors declare that the research was conducted in the absence of any commercial or financial relationships that could be construed as a potential conflict of interest.
